# Electromagnetic Actuation for a Micro/Nano Robot in a Three-Dimensional Environment

**DOI:** 10.3390/mi13112028

**Published:** 2022-11-19

**Authors:** Mostafa Abdelaziz, Maki Habib

**Affiliations:** Robotics, Control and Smart Systems Department (RCSS), School of Science and Engineering, The American University in Cairo (AUC), New Cairo 11835, Egypt

**Keywords:** micro/nano robot, biomedical applications, magnetic field

## Abstract

Micro/nanorobots have several potential biomedical applications, such as drug delivery, minimal invasiveness, and moving within narrow and complex areas. To achieve these desirable applications, precise path tracking and controlling magnetic micro/nanorobots within blood vessels is a crucial but challenging point. In this paper, a three-dimensional electromagnetic actuation system composed of three pairs of Helmholtz coils and three pairs of Maxwell coils is proposed. A closed-loop control algorithm is proposed to enhance trajectory tracking of a micro/nanorobot. Different simulation experiments were carried out using Simulink to verify the performance of the proposed algorithm. Different trajectories were tested in tracking two-dimensional and three-dimensional reference trajectories. The results showed that by using the developed algorithm and electromagnetic actuation system, a micro/nanorobot can follow the desired trajectory within a maximum error of 13 μm.

## 1. Introduction

Biomedical applications have significant potential for micro/nanorobots (MNRs) that operate wirelessly. These untethered, programmable, and powered MNRs can revolutionize conventional treatments, for example, minimally invasive therapies, drug delivery, and focused therapy [1,2,3]. Electromagnetic actuation (EMA) is one of the most successful strategies for gaining access to complicated environments and operating MNRs in a distant and contactless manner [4] by manipulating the magnetic fields around the MNRs [5].

Multiple groups have developed various electromagnetic actuation systems for actuating magnetic MNR within an electromagnetic field.

The two-dimensional EMA system proposed in Ref. [6] is composed of two pairs of Helmholtz coils and two pairs of Maxwell coils. The authors used Helmholtz coils to generate a uniform magnetic field to align the microrobot in the desired orientation and Maxwell coils to generate a uniform gradient magnetic field to propel a microrobot in the aligned controlled direction [6].

Another EMA was proposed, in which a ferromagnetic locomotive microrobot was controlled in three dimensions using typical magnetic resonance imaging (MRI) [7]. The uniform gradient magnetic field generated from an MRI system’s coils actuates and moves a ferromagnetic microrobot with three degrees of freedom (3 DOF) inside a fluidic environment. Using a custom-tailored MRI sequence that repeats image acquisition and actuation gradient, this method can both actuate the microrobot and acquire its position. As the MRI lacks additional coils that provide a magnetic field to align the microrobot in various 3D orientations, the microrobot can only be driven in a limited number of directions. Consequently, the microrobot’s 3D movement is inadequate [7].

The motion control of a five DoFs microrobot (3-DOF position and 2-DOF pointing orientation) was proposed and named the OctoMag electromagnetic system [8]. The OctoMag system consists of eight electromagnetic coils with soft-magnetic cores configured in a specific manner to generate complicated nonuniform magnetic fields [9]. Despite the satisfactory performance of these EMA systems, the workspace is very modest compared to the enormous size of the EMA system. In addition, it is a challenge to calibrate the nonuniform magnetic fields generated by these electromagnets, resulting in construction-related issues [9].

Different EMAs (One Coil, Differential current coil DCC, and combined Helmholtz and Maxwell) were presented and simulated, and the obtained result of the combined Helmholtz and Maxwell coils showed better actuation of the MNR than that of DCC and the one coil EMA, respectively [10].

This paper proposes six-dimensional system (3D for position and 3D for orientation) consisting of three pairs of Helmholtz coils and three pairs of Maxwell coils. The Helmholtz coils are used to produce a uniform magnetic field, while the Maxwell coils are used to generate a uniform magnetic field gradient. The design and configuration of the proposed EMA are described in Section 3. The kinematics, motion control of the magnetic MNR, and the control algorithm are discussed in Section 4. Section 5 shows the simulation results. Finally, the conclusion of the paper is given in Section 6.

## 2. Paper Contribution

The main contribution points of the paper are as follows

- The developed navigation of the MNR describes the pose of the robot in six-dimensions (6D), three dimensions for orientation of an MNR within the *X*, *Y*, and *Z* plane and, three dimensions for position. Three pairs of Helmholtz coils are used for the orientation and the other three pairs of Maxwell coils are used for positioning and MNR in the *X*, *Y*, and *Z* plane. The current supplied to the Helmholtz coils controls the uniform magnetic field generated from these coils to align an MNR to the desired orientation. Moreover, the proposed EMA can actuate the MNR to the desired orientation at any instant;
- The design of the proposed EMA, in terms of the number of turns and coils’ radii, can actuate the small sizes of micro/nanorobots with scales less than 0.2 mm;
- The proposed and designed EMA can actuate and navigate micro/nanorobots within a relatively large region of interest (ROI) 40 × 40 × 40 mm^3^;
- The error in the simulation results for different experiments tests was 13 μm and 8 μm.

## 3. Design of the Driving EMA

The magnetic material is susceptible to magnetic torque and force in a magnetic field. The following equations are used to obtain the magnetic torque (τ) and force (*F*) [11]:

(1) τ=VM×B

(2) $$\tau =\left[\begin{array}{c} \tau_{x}\\ \tau_{y}\\ \tau_{z} \end{array}\right]=V\left[\begin{array}{c} M_{x}\\ M_{y}\\ M_{z} \end{array}\right]\times \left[\begin{array}{c} B_{x}\\ B_{y}\\ B_{z} \end{array}\right]=\left[\begin{array}{ccc} 0 & -M_{z} & M_{y}\\ M_{z} & 0 & -M_{x}\\ M_{y} & M_{x} & 0 \end{array}\right]\left[\begin{array}{c} B_{x}\\ B_{y}\\ B_{z} \end{array}\right]\;$$

(3) F=V(M·∇B)

(4) $$F=\left[\begin{array}{c} F_{x}\\ F_{y}\\ F_{z} \end{array}\right]=V\left[\begin{array}{ccc} M_{x} & M_{y} & M_{z} \end{array}\right]\left[\begin{array}{c} \nabla B_{Mx}\\ \nabla B_{My}\\ \nabla B_{Mz} \end{array}\right]$$

where *V* represents the volume of the MNR, *M* represents its magnetization saturation, and *B* represents magnetic flux.

**Figure 1:**
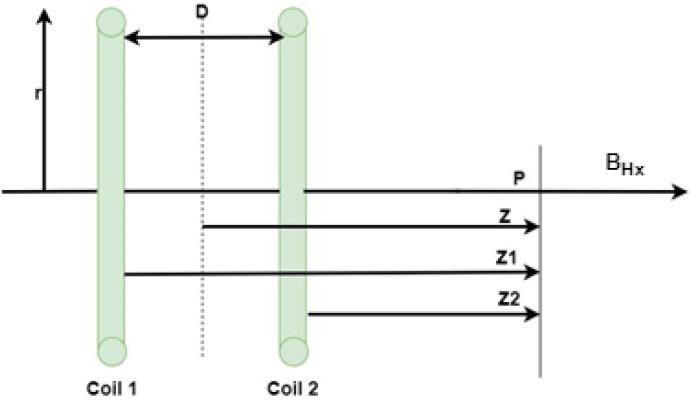
Magnetic field produced from two identical circular coils.

Figure 1 depicts two identical circular coils with the same radius r, separated by a distance D, and supplied with the same amplitude and direction of current. The magnetic field generated by these two circular coils is the total of the individual fields generated by each.

The magnetic field at a selected point P along their central axes with a distance z from the center line between them can be calculated independently for each coil, and the total field can be derived by combining them together. The overall magnetic field at point P will be equal to the sum of the fields of each circular coil, as stated by the following Equation (5) [12]:

(5) $$B_{Hx}=\frac{\mu_{o}N_{Hx}I_{Hx}{r_{Hx}}^{2}}{2}\left(\frac{1}{{\left[{r_{Hx}}^{2}+{\left(\frac{D}{2}-Z_{1}\right)}^{2}\right]}^{3/2}}+\frac{1}{{\left[{r_{Hx}}^{2}+{\left(\frac{D}{2}+Z_{2}\right)}^{2}\right]}^{3/2}}\right)$$

where $B_{Hx}$ is the magnetic flux produced, $N_{Hx}$ is the number of turns, $I_{Hx}$ is the supplied current, $\mu_{o}$ is the permeability of empty space, $r_{Hx}$ is the coil radius, and *D* is the coil-to-coil distance.

**Figure 2:**
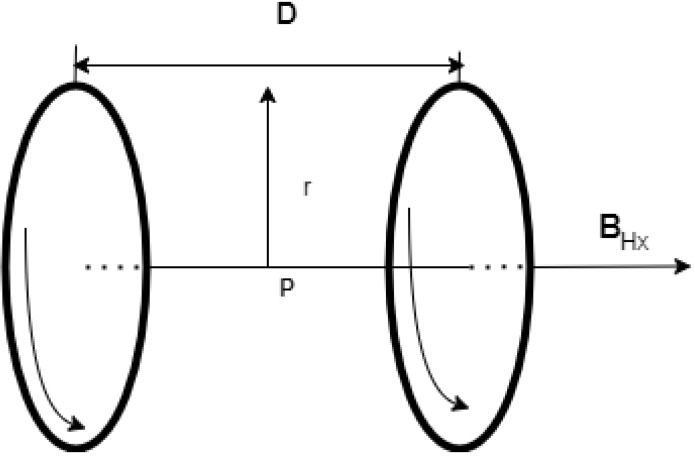
Pair of Helmholtz coils with the same radius and the same applied current direction.

Figure 2 depicts Helmholtz coils invented by Hermann von Helmholtz, which consist of two coils with the same radius that are separated by a distance equal to the coil’s radius. It is employed to produce a uniform magnetic field between the centers of the two coils. The same is applied to the other two axes, *Y* and *Z*.

If the point *p* mentioned in Figure 1 is chosen at the center between the two coils, the distance *z* will be equal to zero; $Z_{1}$ equals D/2 and $Z_{2}$ equals −D/2, respectively. Hence, a new Equation (6) is obtained from Equation (7) as follows [13,14].

(6) $$B_{Hx}=\frac{8}{5\sqrt{5}}\frac{\mu_{o}N_{Hx}I_{Hx}}{r_{Hx}}=k\cdot I_{Hx}$$

where $\mu_{o}$ represents the permeability, $r_{Hx}$ represents the radius of Helmholtz coils, and $N_{Hx}$ represents the number of turns of Helmholtz coils. *k* is a constant representing the geometrical properties of Helmholtz coils [13].

For an MNR to have multidimensional navigation, Helmholtz and Maxwell coils are combined. To enable three-dimensional navigation, three pairs of each are orthogonal sets.

Consequently, the magnetic field produced by three orthogonal Helmholtz coils will be

(7) $$B_{H}=\left[\begin{array}{c} B_{Hx}\\ B_{Hy}\\ B_{Hz} \end{array}\right]=\left[\begin{array}{c} k_{x}I_{Hx}\\ k_{y}I_{Hy}\\ k_{z}I_{Hz} \end{array}\right]$$

**Figure 3:**
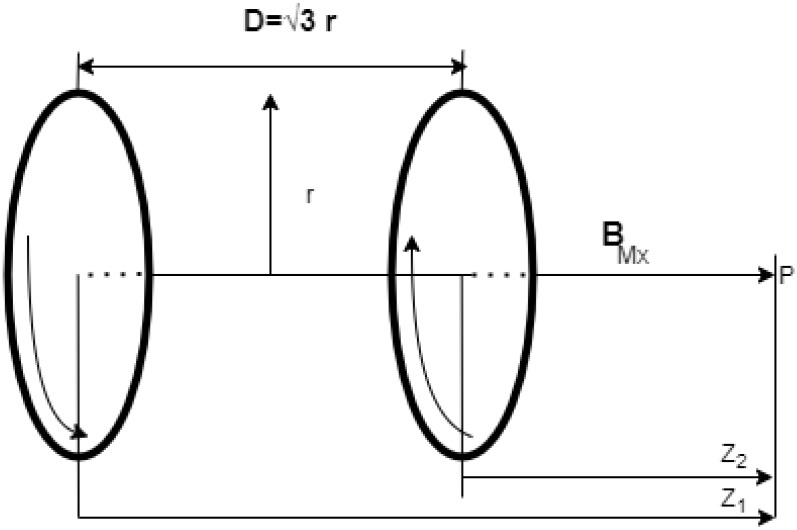
Pair of Maxwell coils with the same radius and opposite applied current direction.

Maxwell coils are an additional configuration. where the distance between the coils is $\sqrt{3}$ times the coils’ radii and the current supplied to the coils is the same magnitude but flows in the opposite direction. In honor of the Scottish physicist James Clerk Maxwell, it bears his name. Along its axis, it is used to generate a uniform gradient of magnetic flux intensity [12,15]. Figure 3 shows a Maxwell coil’s configuration.

(8) $$B_{Mx}=\frac{\mu_{o}N_{Mx}I_{Mx}{r_{Mx}}^{2}}{2}\left(\frac{1}{{\left[{r_{Mx}}^{2}+{\left(\frac{D}{2}-Z_{1}\right)}^{2}\right]}^{3/2}}-\frac{1}{{\left[{r_{Mx}}^{2}+{\left(\frac{D}{2}+Z_{2}\right)}^{2}\right]}^{3/2}}\right)$$

The same is applied to the other two axes *Y*, and *Z*.

(9) $$B_{M}=\left[\begin{array}{c} B_{Mx}\\ B_{My}\\ B_{Mz} \end{array}\right]$$

where $N_{Mx}$ is the number of turns in the Maxwell coil, $I_{Mx}$ is the current supplied to the Maxwell coil, and $r_{Mx}$ is the Maxwell coil radius. From Equation (8), the axial gradient magnetic field produced by Maxwell coils along the x-axis at the center between the coils at point p, where the distance between them (D) is equal $\sqrt{3}$ times the coils’ radii, can be calculated as follows [13,14].

(10) $$\nabla B_{Mxa}=\frac{48}{49}\sqrt{\frac{3}{7}}\frac{\mu_{o}N_{Mx}I_{Mx}}{{r^{2}}_{Mx}}=g_{x}I_{Mx}$$

For actuating an MNR in 3D *(X-Y-Z)* plane, three pairs of Maxwell coils are needed. So, the axial gradient magnetic field will be calculated as in Equation (11).

(11) $$\nabla B_{Ma}=\left[\begin{array}{c} \nabla B_{Mxa}\\ \nabla B_{Mya}\\ \nabla B_{Mza} \end{array}\right]=\left[\begin{array}{c} g_{x}I_{Mx}\\ g_{y}I_{My}\\ g_{z}I_{Mz} \end{array}\right]$$

where $\mu_{o}$ represents the permeability, $r_{Mx}$ represents the radius of Maxwell coils, and $N_{Mx}$ represents the number of turns of Maxwell coils. $I_{Mx}$ is the current supplied to Maxwell coils, $g_{x}$ is a constant representing the geometrical properties of Maxwell Coils [13].

Equation (11) expresses the axial component of the magnetic field gradient produced by Maxwell coils along the x-axis. The radial component of the gradient magnetic field produced by Maxwell coils along the x-axis is half of the axial component in magnitude and in the opposite direction [13].

(12) $$\nabla B_{Mxr}=-\frac{1}{2}\nabla B_{Mxa}$$

**Figure 4:**
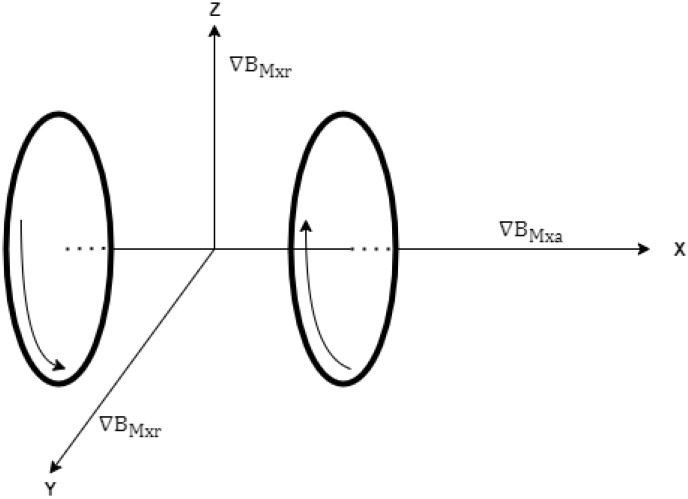
Axial and radial components of the gradient magnetic field.

Figure 4 shows the axial gradient magnetic field generated along x-axis Maxwell coils and its radial components along *Y* and *Z* axes.

The gradient magnetic field produced from three orthogonal pairs of Maxwell coils is shown below in Equation (13).

(13) $$\nabla B_{M}=\left[\begin{array}{c} \nabla B_{Mx}\\ \nabla B_{My}\\ \nabla B_{Mz} \end{array}\right]=\left[\begin{array}{c} g_{x}I_{Mx}-0.5g_{y}I_{My}-0.5g_{z}I_{Mz}\\ g_{y}I_{My}-0.5g_{x}I_{Mx}-0.5g_{z}I_{Mz}\\ g_{z}I_{Mz}-0.5g_{y}I_{My}-0.5g_{x}I_{Mx} \end{array}\right]$$

**Figure 5:**
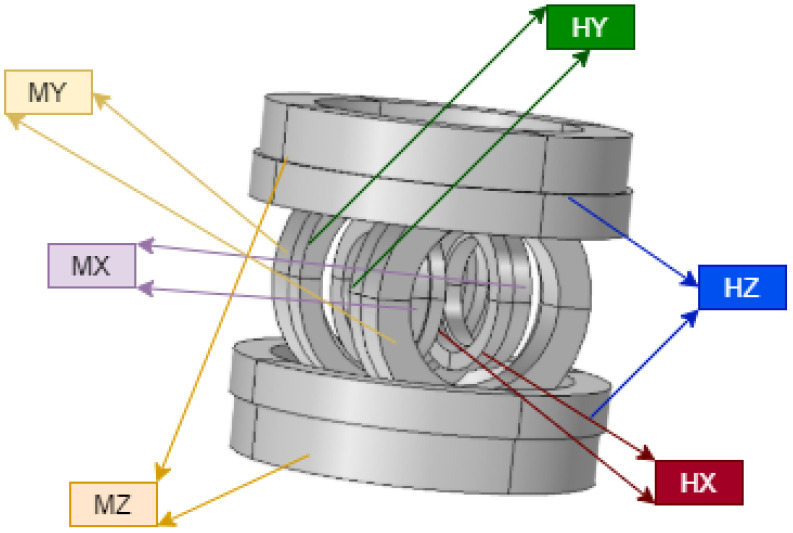
EMA system consisting of three pairs of Helmholtz coils and three pairs of Maxwell coils.

To achieve MNR actuation control under the influence of a magnetic field, 3D EMA coil system is required to generate a controllable magnetic field in 3D space. By adjusting the currents that flow through the coils, it is possible to control the magnetic field produced by the coils, and thus the movement of the magnetic MNR. This idea motivates us to utilize Helmholtz coils and Maxwell coils to create a 3D EMA system consisting of three sets of Helmholtz coils and three sets of Maxwell coils arranged in three orthogonal axes. Helmholtz coils are responsible for the orientation of the MNR, while the Maxwell coils are responsible for the actuation and moving of the MNR. The main parameters of the proposed EMA are listed in Table 1:

The proposed EMA shown in Figure 5 provides a uniform magnetic field along the region of interest (ROI) with volume coverage yields to (40 × 40 × 40) mm^3^ produced from the Helmholtz coils, and a uniform magnetic gradient along the region of interest produced from the Maxwell coils [10].

## 4. Actuation Control Algorithm of the MNR with the H&M EMA System

The MNR locomotion is affected in the vertical axis by two forces, its gravitational weight $F_{g}$ and the fluid buoyancy force $F_{b}$. Drag force $F_{d}$ also resists the motion of an MNR, negatively resulting from the viscosity of the fluid. Additionally, the driving magnetic force $F_{m}$ actuated the MNR. The dynamic equation of the MNR for any given point p in the 3D ROI is provided by the following Equation (14):

(14) $$F_{m}+F_{d}+F_{g}+F_{b}=ma=m\ddot{p}$$

The microrobot is typically placed in a liquid with a low Reynolds number, and, consequently, its inertial force can be neutralized. Therefore, the drag force $F_{d}$ can be calculated for an MNR moving in a stagnant fluidic environment as follows:

(15) $$F_{d}=6\pi \eta Rv$$

such that η is the fluid’s dynamic viscosity, *R* is the MNR’s radius, and *v* is the MNR’s speed. The drag force is not a constant value, since it depends on the speed of the MNR, which depends on the intensity of the magnetic field gradient.

Both the gravitational and buoyancy forces are constant.

(16) $$F_{g}=V\rho g$$

(17) $$F_{b}=V\rho_{f}g$$

The resultant of both gravitation force $F_{g}$ and buoyancy force $F_{b}$ are calculated as in the following equation:

(18) $$F_{g}-F_{b}=V\left(\rho -\rho_{f}\right)g$$

where ρ is the density of the MNR, *V* is the MNR’s volume, $\rho_{f}$ is the density of the fluid, and *g* is the gravity constant.

**Figure 6:**
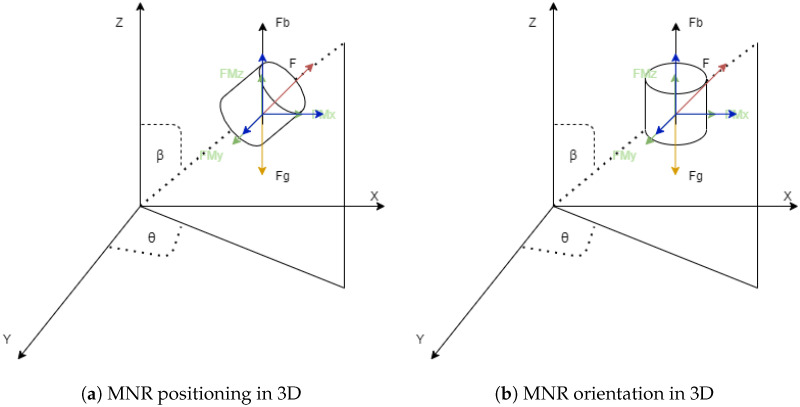
MNR locomotion and orientation in 3D.

Since the proposed magnetic actuation systems are based on Helmholtz and Maxwell coil configurations, which can generate a uniform magnetic field and uniform magnetic gradients, respectively, this section’s analysis is crucial. It will be explained in the following section how this can be used to control MNRs. We will assume that the MNR is located in any plane in the three dimensional space, with the angle β in the positive z-axis, as shown in Figure 6. Figure 6b shows the orientation of the MNR produced from the uniform magnetic field generated by Helmholtz, while Figure 6a shows the position and propelling of the MNR.

Equation (1) demonstrates the torque required to align an MNR to the desired location when using Helmholtz coils. The resulting magnetic field generated by two pairs of Helmholtz coils aligns the MNR to the desired orientation θ within the x-y plane such that tan θ = $B_{H,y}$/$B_{H,x}$.

(19) $$tan\theta =\frac{B_{Hy}}{B_{Hx}}=\frac{K_{y}I_{Hy}}{K_{x}I_{Hx}}$$

So, if both $k_{y}$ and $k_{x}$ have the same value, the coil currents can be adjusted to align the MNR to the desired orientation within the x-y plane.

In order to extend the dimensionality from 3D to 6D, another pair of Maxwell coils is added to represent the position in the Z-axis as well as a pair of Helmholtz coils to generate an additional angle, which will help in combination to conclude the six dimensions. Another angle β is defined, which is calculated as follows:

(20) $$tan\;\beta =\frac{B_{Hz}}{\sqrt{{B_{Hx}}^{2}+{B_{Hy}}^{2}}}$$

For the purpose of generating the desired orientation in the x, y z plane, adjusting the currents applied to the three pairs of Helmholtz coils, the desired magnetic field will be generated, resulting in aligning the MNR to the desired orientation in the x, y, and z plane. Therefore, the desired magnetic field B to align the orientation can be obtained as follows:

(21) $$\left\{\begin{array}{c} B_{Hx}=B_{H}sin\;\theta sin\;\beta \\ B_{Hy}=B_{H}cos\;\theta sin\;\beta \\ B_{Hz}=B_{H}cos\;\beta \end{array}\right.$$

Maxwell coils produce a uniform magnetic gradient that generates a propulsion force, which is expressed in Equation (13).

The magnitude of the MNR’s total magnetization saturation *M* is constant, so the components of the magnetization is described as follows:

(22) $$M=\left[\begin{array}{c} M_{x}\\ M_{y}\\ M_{z} \end{array}\right]$$

(23) $$\left\{\begin{array}{c} M_{x}=Msin\;\theta sin\;\beta \\ M_{y}=Mcos\;\theta sin\;\beta \\ M_{z}=Mcos\;\beta \end{array}\right.$$

Since both the volume and the magnetization of the MNR are known, the three components of the magnetic driving force are derived as follows:

(24) $$\left\{\begin{array}{c} F_{x}=M_{x}Vg_{x}=MVg_{x}sin\;\theta sin\;\beta \\ F_{y}=M_{y}Vg_{y}=MVg_{y}cos\;\theta sin\;\beta \\ F_{z}=M_{z}Vg_{z}=MVg_{z}cos\;\beta \end{array}\right.$$

Such that $g_{x}$, $g_{y}$, and $g_{z}$ are the components of the magnetic gradient. To actuate and propel the MNR in the desired orientation θ, the ratio between the propulsion forces in the x and y directions must be equal to tan θ, such that:

(25) $$\frac{F_{x}}{F_{y}}=tan\;\theta =\frac{MVg_{x}sin\;\theta sin\;\beta }{MVg_{y}cos\;\theta sin\;\beta }$$

So, from Equation (25), any two pairs of Maxwell coils generate the same magnetic field gradient. Consequently, $g_{x}$ = $g_{y}$ is deduced. In addition, from now on, $g_{m}$ is used instead of $g_{x}$ and $g_{y}$.

Since both the gravity and buoyancy forces in the z direction are constant, $F_{z}$ is divided into two parts, $F_{zg}$ and $F_{zd}$, where $F_{zg}$ compensates for the gravity and buoyancy forces and $F_{zd}$ is the magnetic driving force in the z-axis. The magnetic flux gradient $g_{z}$ is also separated into $g_{zg}$, representing the magnetic flux gradient produced to compensate for the gravitational force and $g_{zd}$ is the magnetic gradient for driving the MNR. The following equations are deduced [16,17].

(26) $$\frac{F_{zd}}{\sqrt{{F_{x}}^{2}+{F_{y}}^{2}}}=cot\;\beta =\frac{MVg_{zd}cos\;\beta }{MVg_{m}sin\;\beta }=cot\;\beta \frac{g_{zd}}{g_{m}}$$

(27) $$F_{zg}=V\left(\rho -\rho_{f}\right)g=F_{g}-F_{b}$$

From Equation (26), it can be deduced that to align an MNR to the desired orientation (β), $g_{zd}$ = $g_{m}$. While from Equation (27), $F_{zg}$ is calculated and kept as a constant value and the current $I_{mz}$ is divided into two parts, one responsible for generating $F_{zg}$ to hold and lock the MNR at any position in the z axis, and the other part responsible for ($g_{zd}$) $F_{zd}$ to actuate the MNR in a vertical position.

## 5. Simulation Results

The proposed system is intended for use in biomedical applications such as blood vessel navigation. The MNR is constructed of Neodymium (N52). The MNR has a cylindrical shape with a 1.5 mm diameter and length of 2 mm. The parameters of the MNR robot are depicted in Table 2. The ROI is filled with a viscous fluid, which is silicon oil with kinematic viscosity (350cs). The value of the magnetic field generated from Helmholtz coils and the value of the magnetic field gradient from Maxwell coils are obtained from previous work [10,18].

By regulating the position of the MNR, the control architecture relies on a closed-loop feedback system using the PID controller. The reference input is the desired position, i.e., MNR’s desired coordinate. A set of coordinates are provided to the system in order to attain the desired trajectory. The position error serves as the controller’s input. The output of the PID controller aims to manipulate the current for the Maxwell pairs of coils.

The control sequence is as follows:

- First, extract the coordinates ($x_{r}$, $y_{r}$, $z_{r}$) of the desired trajectory at the current time instant.
- Calculate the required orientation angles θ and β for each coordinate as follows:
  (28) $$tan\theta =\frac{y_{r}}{x_{r}}$$
  (29) $$cot\;\beta =\frac{z_{r}}{\sqrt{{x_{r}}^{2}+{y_{r}}^{2}}}$$
- Calculate the currents supplied to the Helmholtz coils $I_{Hx}$, $I_{Hy}$, and $I_{Hz}$.
- The PID controller issues a control action that manipulates the input current of the Maxwell coils. The PID controller parameters are obtained using the genetic algorithm optimization technique. The values of the PID parameters are the proportional ($K_{p}$), which is 6, the integral ($K_{i}$), which is 0.01, and ($K_{d}$), which is 6.

**Figure 7:**
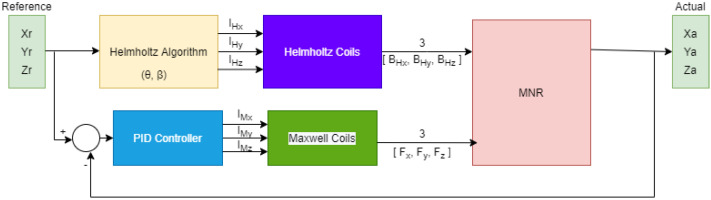
Block diagram representing the actuation control algorithm.

This algorithm is represented as a block diagram, which is illustrated in Figure 7.

Simulation tests were carried out to ensure the response of the PID controller and the EMA system to actuate an MNR within the desired trajectory. Two examples are considered to demonstrate the following two different trajectories by the developed system.

The first example is following a diamond trajectory in 2D (x-y plane) with corner coordinates (x,y): (0,0)-(1,1)-(2,0)-(1,−1) and finally to the starting point (0,0).

**Figure 8:**
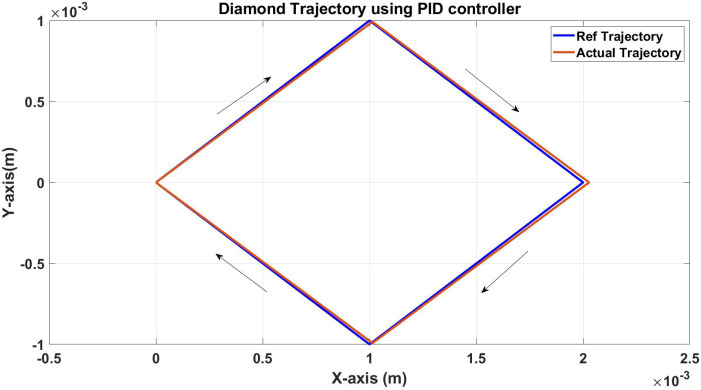
Simulation results of the diamond trajectory (reference and actual) using the PID controller.

**Figure 9:**
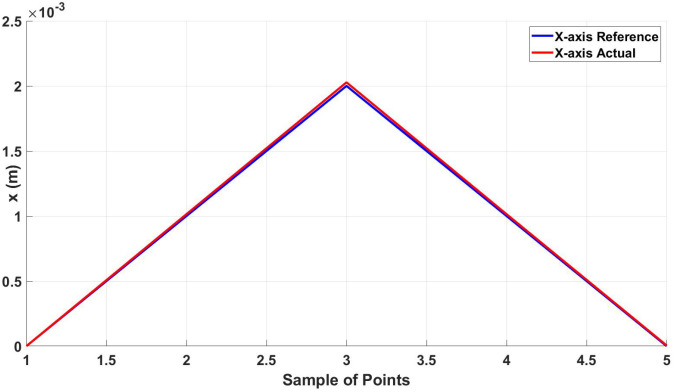
Actual and reference position of the diamond trajectory in the x-axis.

**Figure 10:**
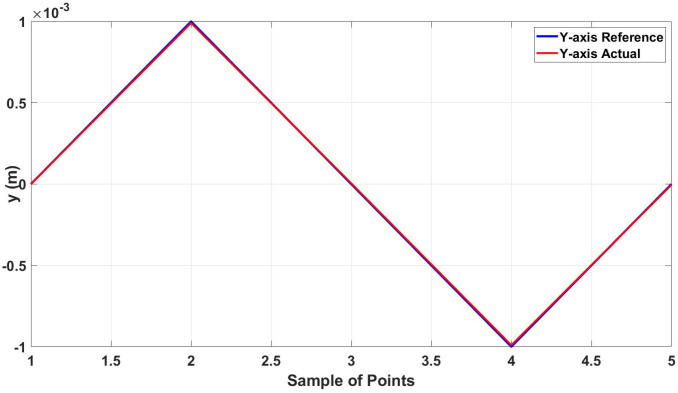
Actual and reference position of the diamond trajectory in the y-axis.

As shown in Figure 8, the actual trajectory closely follows the desired one with an error between the two trajectories of 13.011 μm in the x-axis and 8*μ* in the y-axis. Figure 9 and Figure 10 show the actual and reference of both x and y coordinates, respectively.

**Figure 11:**
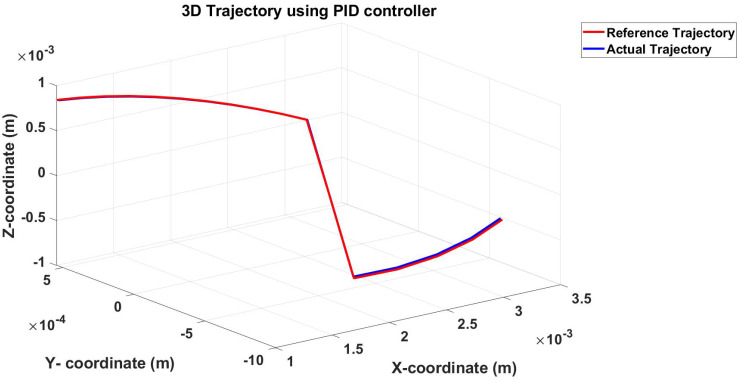
Simulation results of the 3D trajectory (reference and actual) using the PID controller.

The second example is following a 3D trajectory, and the response of the controller and the EMA system is shown in Figure 11.

**Figure 12:**
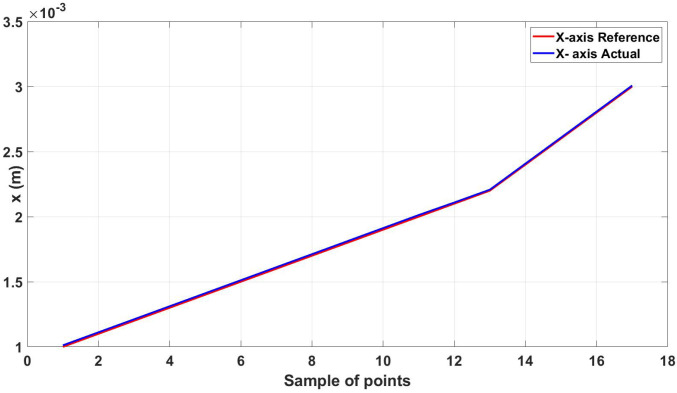
Actual and reference position of the diamond trajectory in the x-axis.

**Figure 13:**
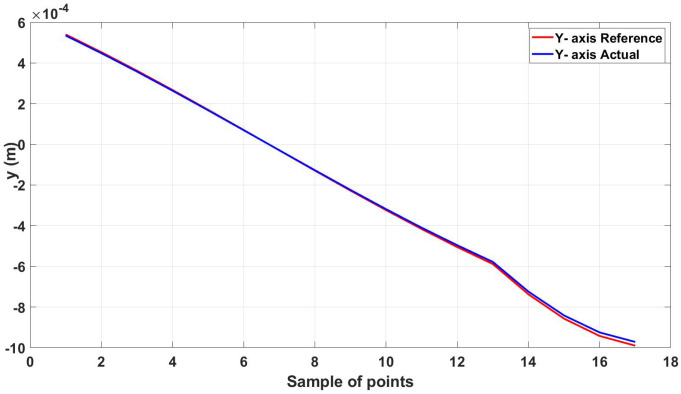
Actual and reference position of the diamond trajectory in the y-axis.

**Figure 14:**
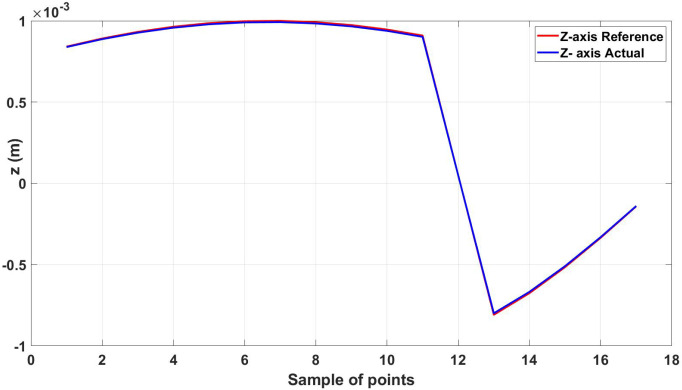
Actual and reference position of the diamond trajectory in the z-axis.

The root mean square error for the three axes was calculated for the trajectory in Figure 11, shown in Table 3. Figure 12, Figure 13 and Figure 14 show the actual and reference $x_{r}$, $y_{r}$, and $z_{r}$ coordinates.

**Figure 15:**
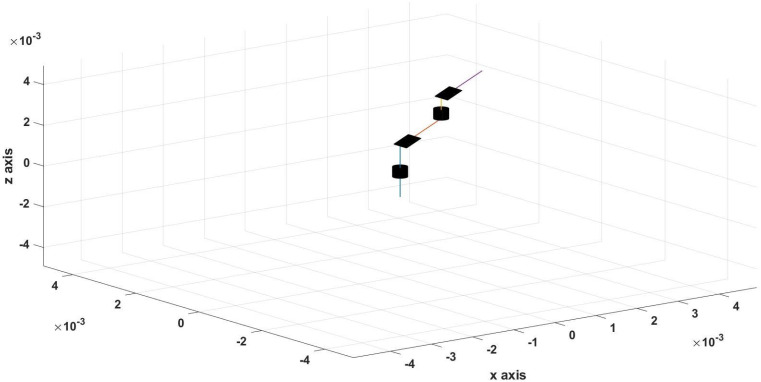
Orientation of an MNR in 3D.

A simulation test was carried out to check the ability of the proposed EMA to orient the MNR at any position, and the result is shown in Figure 15. It can be deduced that MNR can be oriented at any instant to the desired one. This test validates the ability of the proposed EMA to actuate the MNR. In addition, the MNR can stand still and at any position and have different orientations.

## 6. Conclusions

In this paper, we introduced a three-dimensional (3D) system for position and a 3D system for orientation to control an MNR by developing an EMA consisting of three pairs of Helmholtz coils and three pairs of Maxwell Coils. The motion analysis of the MNR in both position and orientation was conducted and discussed. PID-based closed loop control technique with tuned parameters was used to improve EMA position tracking along a desired trajectory. Simulation tests were carried out to test the developed system. The results show that MNR can navigate and track its position along a desired trajectory with a maximum error of 13 μm. In addition, the simulation results confirm the ability of the closed loop EMA system to actuate an MNR as required. Future work is currently undergoing to experimentally validate the simulation results.

## Figures and Tables

**Table 1:** Main parameters of the proposed EMA coils.

Coil	Radius (mm)	No. of Turn N
X-axis Helmholtz Coil (HX)	50	100
X-axis Maxwell coil (MX)	45	130
Y-axis Helmholtz Coil (HY)	80	145
Y-axis Maxwell coil (MY)	70	400
Z-axis Helmholtz Coil (HZ)	125	600
Z-axis Maxwell coil (MZ)	112	1250

**Table 2:** MNR parameters.

MNR diameter	1.5 mm
MNR length	2 mm
MNR density (ρ)	7500 Kgm^−3^
MNR mass (m)	26.55 μKg
Magnetization saturation (M)	10${}^{6}$ A/m
MNR volume (V)	3.54 mm^3^
Fluid density ($\rho_{f}$)	968 Kgm^−3^
Permeability ($\mu_{o}$)	4$\pi *10^{-7}\;{N/A}^{2}$
Gravitational force ($F_{g}$)	265 μN
Buoyancy force ($F_{b}$)	34.2 μN
$k_{x}$	1.66 mT/A
$k_{y}$	1.63 mT/A
$k_{z}$	4.3 mT/A
$g_{x}$	0.046 T/mA
$g_{y}$	0.048 T/mA
$g_{z}$	0.08 T/mA

**Table 3:** RMS error following the 3D trajectory.

Axis	RMS Error
x error	10 μm
y error	9 μm
z error	6 μm

## Data Availability

Not applicable.

## Abbreviations

The following abbreviations are used in this manuscript:

EMA	Electromagnetic Actuation System
MNR	Micro/nano robot
ROI	Region of Interest
